# Tracing and characterization of foodborne botulism caused by the new MLST type *Clostridium botulinum* A2 in Hebei province, China

**DOI:** 10.3389/fmicb.2025.1567360

**Published:** 2025-03-26

**Authors:** Ziwei Lu, Jinzhi Feng, Xia Luo, Hui Sun, Ying Huang, Shuangshuang Lu, Dai Wang, Xuefang Xu, Xuancheng Lu, Lixia Xu

**Affiliations:** ^1^National Key Laboratory of Intelligent Tracking and Forecasting for Infectious Diseases (NITFID), Chinese Center for Disease Control and Prevention, Beijing, China; ^2^State Key Laboratory of Vaccines for Infectious Diseases, Xiang An Biomedicine Laboratory, National Innovation Platform for Industry-Education Integration in Vaccine Research, School of Public Health, Xiamen University, Xiamen, China; ^3^National Key Laboratory of Intelligent Tracking and Forecasting for Infectious Diseases, National Institute for Communicable Diseases Control and Prevention, Chinese Center for Disease Control and Prevention, Beijing, China; ^4^Quzhou Maternal and Child Health Care Hospital, Quzhou, China

**Keywords:** *C. botulinum*, foodborne botulism, BoNT, subtype A2, genome reduction

## Abstract

Foodborne botulism caused by botulinum neurotoxin (BoNT) remains an important form of botulism worldwide, with a high mortality rate and prolonged hospitalization time. *Clostridium botulinum* (*C. botulinum*) is the main microorganism responsible for producing BoNTs. This study reports a case of foodborne botulism caused by a *C. botulinum* subtype A2 strain from pickled eggs. We detected the BoNT gene using real-time PCR and the BoNT through the mouse bioassay (MBA) from both the patient’s feces and the pickled eggs and isolated *C. botulinum* A strains. The genetic SNP and phylogenetic tree analysis confirmed that the *C. botulinum* strains from the patient’s feces and the pickled eggs had the same origin. Although Hebei province is a high-incidence area for foodborne botulism, this is the first reported case of botulism caused by *C. botulinum* type A in pickled eggs in this region. The 10 isolated *C. botulinum* A strains all had a new ST193 type and contained the BoNT A toxin-producing gene and a potential virulence factor, GroEL. The BoNT A gene was classified as subtype A2 and belonged to the orfx cluster. The antibiotic resistance genes identified included *cfr*, *spw*, and *vat*. We also found that the genomic size of *C. botulinum* in the feces was smaller than that in the food and that most of the missing genes were related to desiccation/radiation resistance proteins, which might indicate gene loss during the process of entering the intestine. For this foodborne botulism outbreak, more emphasis should be placed on promoting food safety awareness among residents to prevent such botulism outbreaks in the future.

## 1 Introduction

Botulism is a serious paralytic illness caused by botulinum neurotoxin (BoNT), mainly produced by bacteria such as *Clostridium botulinum* (*C. botulinum*), *Clostridium butyricum* (*C. butyricum*), and *Clostridium baratii* (*C. baratii*) (Mad’arova et al., 2017). There are five forms of botulism—foodborne botulism, infant botulism, iatrogenic botulism, adult botulism, and wound botulism (Cenciarelli et al., 2019). Foodborne botulism is the predominant form of poisoning worldwide. It refers to the contamination of food with botulinum spores during the production process, incomplete sterilization after manufacturing, germination and reproduction of spores in anaerobic environments, toxin production, ingestion of the pre-formed toxin by organisms through the gastrointestinal tract, absorption into the circulatory system, and the resulting botulism (Mad’arova et al., 2017; Meurice et al., 2023; Tran et al., 2024; Hamad et al., 2023; Oliveira et al., 2022; Negrut et al., 2021). The incubation period ranges from 2 h to 10 days, typically 12 to 72 h. Initial symptoms include nausea, vomiting, and diarrhea, followed by headache, dizziness, general fatigue, and specific signs of botulism, such as bulbar paralysis, which manifests as diplopia, dysarthria, dysphonia, and dysphagia—commonly referred to as the “4D” symptoms (Koepke et al., 2008; Trehard et al., 2016). Other manifestations include drooping eyelids, dilated pupils, facial paralysis with a lack of expression, and difficulty opening the mouth and protruding the tongue. However, consciousness is usually clear, and there is no fever or sensory impairment (Lafuente et al., 2013; Rosen et al., 2020).

Foodborne botulism continues to account for the majority of botulism cases reported in China (Zhang et al., 2010; Lim et al., 2019; Ma et al., 2022). In 2023, a severe outbreak of type B botulism was caused by canned sardines (Meurice et al., 2023). It is mainly caused by homemade food, with high-risk items including soy products (stinky tofu, fermented tofu, soybean paste, dried tofu, soy sauce, *etc*.), meat products (dried beef, dried yak beef, cured duck gizzard, rabbit meat products, *etc*.), fish products (cured fish, vacuum-packaged fish, *etc*.), egg products (pickled eggs), honey, and other cured high-protein foods (Zhang et al., 2010; Shih and Chao, 1986; Wang, 1986; Dong et al., 2022). The storage method of vacuum-packaged food in modern industry creates favorable conditions for the growth of anaerobic bacteria, contributing to the recent increase in cases of foodborne botulism in China. The Xinjiang Uygur Autonomous Region in China is a high-incidence area for traditional foodborne botulism, which is believed to be related to the widespread presence of *C. botulinum* spores in the soil (Ma et al., 2022; Hu and Tong, 1988; Li H. et al., 2022). In addition to Xinjiang, other provinces and regions such as Shandong, Hebei, Henan, Qinghai, Tibet, and Shanxi are also prone to foodborne botulism.

The reported cases of foodborne botulism in this study originated from Hebei, a high-incidence area, and were caused by the high-risk food—homemade pickled eggs. The homemade pickled eggs in this study were processed as follows: The boiled eggs were peeled and placed in a plastic container with salt. The container was sealed and stored at room temperature for 6 days. Afterward, more salt was added to the container, and the eggs continued to be stored at room temperature for another 10 days with the seal intact. *C. botulinum* a strains were isolated from both the homemade pickled eggs and the patients’ feces, confirming the source of intoxication. The toxicity, genomic sequence, toxin sequence, other virulence gene factors, and drug resistance gene characterization of the isolated strains were analyzed. The genetic SNP analysis of the strains isolated from the food and patients confirmed that they originated from the same source.

## 2 Case report

By the end of July 2024, Patient A had made a batch of homemade pickled eggs, as mentioned above. The specific process involved boiling the eggs, peeling them, adding salt, and storing them in a sealed plastic container at room temperature for 16 days. Patient B ate a small piece of the egg yolk, while Patient A consumed the rest of the egg. Five days after eating the eggs, Patient A experienced mild weakness in the limbs and recovered without special treatment. One week after consuming the eggs, Patient B exhibited symptoms such as vomiting, mental impairment, drowsiness, ptosis, ophthalmoplegia, limited mouth opening, and gradually decreasing muscle strength in all limbs and went to the hospital. On the third day of admission, Patient B’s condition worsened, with respiratory muscle involvement, slight dyspnea, a weak voice, a fast heart rate, and blood gas analysis indicating CO_2_ retention. Invasive respiratory support with endotracheal intubation and mechanical ventilation was provided. Due to the difficulty of clinical diagnosis, samples were not collected in time. Samples of the pickled eggs and feces from Patient A (after 18 days from taking pickled eggs) and Patient B (after 7 days from admission) were collected and sent to the laboratory of the Chinese Center for Disease Control and Prevention for testing. The laboratory test results were reported back to the hospital within 2 days, but no antitoxin was used as Patient B showed improvement after assessment. Patient B continued to improve after 8 days of hospitalization and was discharged after 14 days.

## 3 Materials and methods

### 3.1 Enrichment and strain isolation

The protocol for *C. botulinum* strain isolation was developed according to the Microbiological Examination of Food Hygiene and the Examination of *C. botulinum* and Botulinum Toxin (National Standard of the People’s Republic of China GB/T 4789.12–2016). A total of 20 g of the pickled eggs or feces was added to a gelatin buffer solution, and the supernatant was used for enrichment. All culture tubes were placed in an anaerobic glove box for 5 days at 35°C. Then, the enriched inocula were plated onto egg yolk agar and incubated at 35°C for 2 days until colonies formed. Pure culture was checked using gram staining, PCR for 16S rRNA, the mouse bioassay (MBA), and genomic sequencing.

### 3.2 Real-time PCR for BoNT gene detection

The pickled eggs, feces, and enriched inocula were tested using real-time PCR for quick diagnosis. DNA was extracted using the QIAamp Power Fecal Pro DNA Kit (Germany). The method was carried out as described by Gao et al. (2022). A Ct value ≤35 was considered positive.

### 3.3 Mouse bioassay (MBA)

The MBA was performed as previously described (Xin et al., 2019). Briefly, 15–20 g of ICR female mice were used in this study. Pickled eggs or feces samples were prepared as mentioned above. Culture supernatants were processed as described by Xin et al. (2019). The animal experiments conformed to China’s Regulations for Experimental Animals and received approval from the Institutional Animal Care and Use Committee of the Chinese Center for Disease Control and Prevention for all procedures involving animal care and experimentation.

### 3.4 DNA extraction and genomic sequencing

Cultures of *C. botulinum* were collected, and genomic DNA was extracted using the QIAamp DNA Mini Kit (Germany) according to the manufacturer’s instructions. Sequencing libraries were generated using the NEBNext® Ultra™ DNA Library Prep Kit for Illumina (NEB, USA) following the manufacturer’s recommendations, and index codes were added to attribute sequences to each sample. The whole genome of *C. botulinum* was sequenced using Illumina NovaSeq PE150 at Beijing Novogene Bioinformatics Technology Co., Ltd.

### 3.5 Processing of whole genome sequencing data

Sequencing data quality control, assembly, and gene prediction/annotation were performed using FastQC, SPAdes v3.13.1, and Prokka v1.14.6, respectively. MUMmer 3.23 was employed to identify SNPs among all bacterial genomes and perform merging. The whole-genome single nucleotide polymorphism (wgSNP) data were used to construct the evolutionary tree. Gene prediction results were obtained using the Prokka software and compared with the virulence factor database (VFDB) and the Comprehensive Antibiotic Resistance Database (CARD, ResFinder, ARGANNOT) using the BLAST software to analyze the presence of virulence genes and antibiotic resistance genes in the strains.

The sequencing reads of the 10 isolates were separately mapped to the reference genomes representing most serotypes of *C. botulinum* (Loch Maree, Hall 183, Langeland, H114590007, H111880801g, NCTC 9837, NCTC 8266, CDC 1690, VPI 7943, and Prevot 1,542), and high-quality SNPs were subsequently identified using CLC Genomics Workbench (Version9.0). A phylogenetic tree was also constructed based on the sequences of seven *C. botulinum* genes (*aroE*, *mdh*, *aceK*, *oppB*, *rpoB*, *recA*, and *hsp*) (Halpin et al., 2021; Jacobson et al., 2008a) and the BoNT gene.

### 3.6 Data availability

The sequencing data were deposited in the NCBI under BioProject accession number PRJNA1203909 and submission number SUB14938036.

## 4 Results

### 4.1 Isolation of the *C. botulinum* strains and toxin (gene) detection

The pickled eggs and feces from Patients A and B were tested using the MBA for toxin, real-time PCR for toxin gene analysis, and enrichment for *C. botulinum* strains isolation. Toxin A was detected using the MBA in both the food and Patients A and B’s feces (pickled eggs) (Table 1). The toxin A gene was also detected using real-time PCR in the pickled eggs and Patients A and B’s stool. A total of 10 *C. botulinum* strains were isolated, with nine strains from the food and one strain from Patient A’s feces (Table 1). The toxin A gene and toxin A were also detected in these strains using real-time PCR and the MBA, respectively. The neurotoxin A gene was further confirmed using genomic sequencing.

**Table 1:** Samples and test results.

Sample	MBA	Real-time PCR	Isolated strains	Strain ID
Pickled eggs	A	A	*C. botulinum* A	HBFD001-006, 008–010
Feces from Patient A	A	A	*C. botulinum* A	HBFEX01
Feces from Patient B	A	A	–	–

### 4.2 Pangenomic analysis and SNP typing

The whole genome size of these 10 *C. botulinum* strains was approximately 4.0 M, containing 3,770 to 3,776 CDS, with HBFEX01 from the feces having the fewest CDS. HBFD03 contained four more CDS than HBFEX01. Except for HBFD03, each strain from the food had two more CDS compared to HBFEX01 from the stool. All these differential CDS were hypothetical proteins, but most of them were around CDS coding desiccation/radiation resistance proteins. The GC content was approximately 28.1%, which is consistent with previous *C. botulinum* strains. The *bont* gene was located on the chromosome in the 10 *C. botulinum* strains. No plasmids were observed in the obtained sequences.

**Figure 1:**
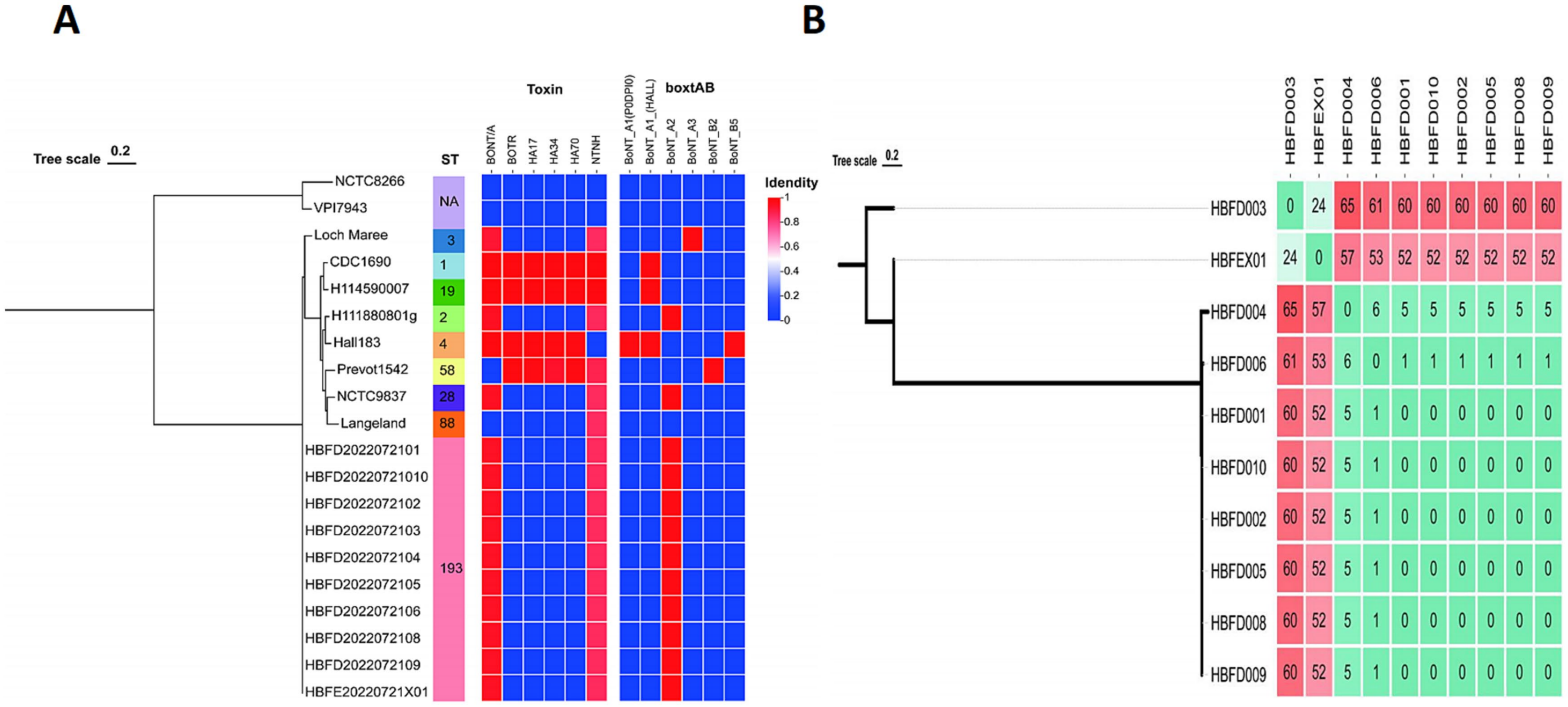
**(A)** Phylogenetic trees showing the relationships between the *C. botulinum* genomes based on the wgSNP, ST type, and neurotoxin genes. **(B)** The wgSNP cluster diagram of the isolated strains showing the number of SNPs between the two strains.

To trace the source of this botulism outbreak, we analyzed the 10 isolated strains alongside 10 reference genomes, including Group I and Group II strains, on the basis of the wgSNP. These 10 reference strains included serotypes A, B, E, and F, which are mainly responsible for human botulism. A total of 10 isolated strains from the feces and food were classified as Group I strains and formed a monophyletic branch on the tree (Figure. 1A). This indicated that the strains isolated in this study belonged to Group I, exhibiting their own evolutionary characteristics.

To further analyze the correlation between each strain from the feces and food, we constructed a phylogenetic tree for the 10 isolated strains separately based on the wgSNP. The strains HBFD004, 006, 001, 010, 002, 005, 008, and 009 formed a single phylogenetic unit, with 0 SNP among the HBFD001, 010, 002, 005, 008, and 009 strains (Figure 1B). HBFEX01 from the feces had the fewest SNPs, with HBFD003 showing 24 SNPs between them, indicating a closer distance on the phylogenetic tree. All of the 24 SNPs were intergenic mutations, except for three SNPs located in the gene coding regions. Of the three SNPs located in the coding region, only one had an amino acid mutation, which was a conservative amino acid replacement. The mutated SNP was located in *cbiH* at p.T136A. The amino acid changed from T to A due to the mutation of the base from A to G.

### 4.3 Botulinum neurotoxin types and MLST determination

**Figure 2:**
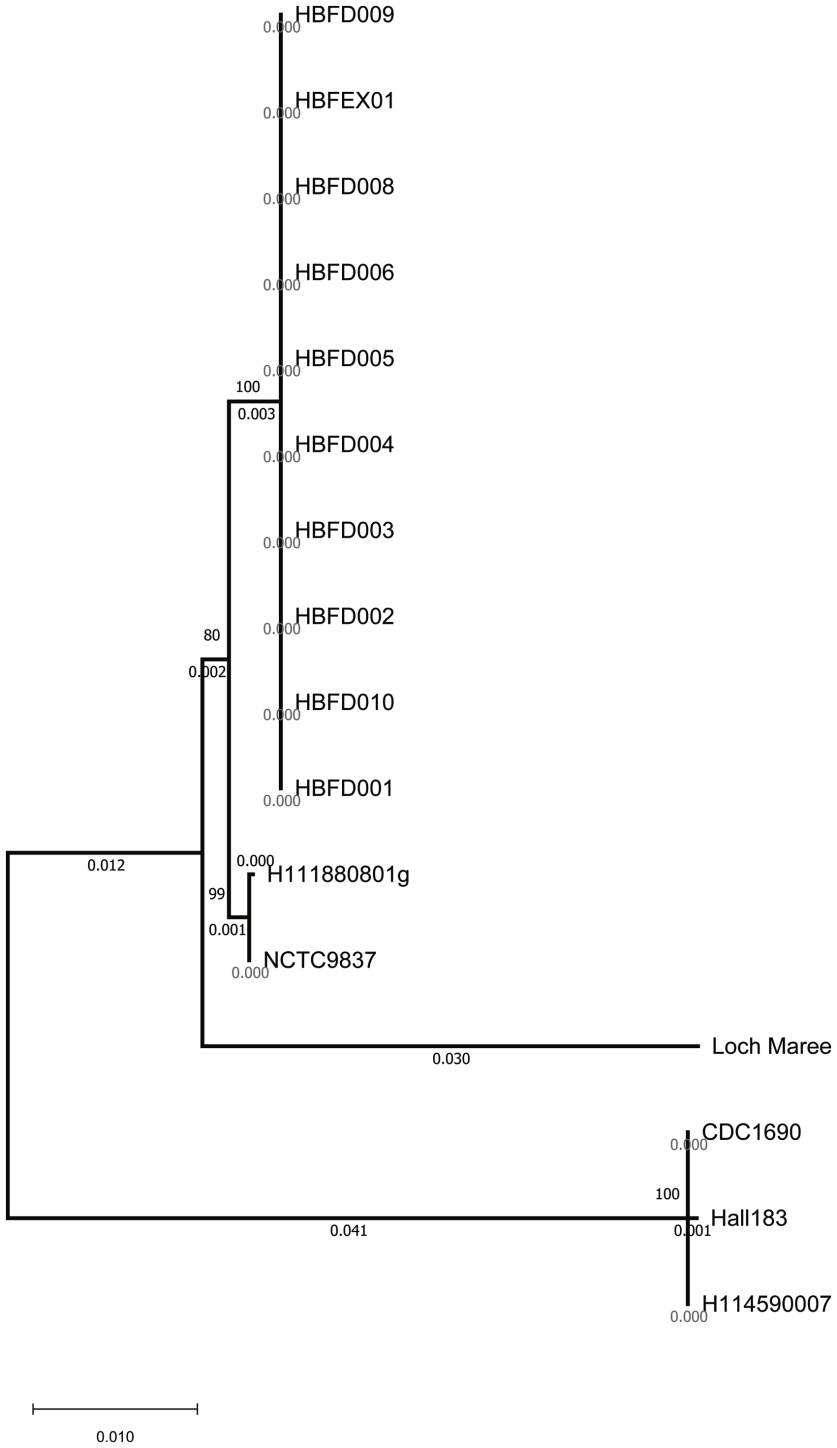
Clustering tree of the *bont* gene nucleic acid sequences using the neighbor-joining method.

To investigate the most important virulence factor—neurotoxin—in these strains, the *bont* genes were analyzed and compared. Although the 10 strains showed significant differences at the whole genome level with the referred A2 strains on the phylogenetic tree, the *bont* genes of the 10 isolated strains were all classified as the A2 subtype (Figures 1A, 2). The similarity among the nucleic sequences of these 10 strains was 100%. The evolutionary relationship of the *bont* A gene sequences of these strains with other A subtypes is shown in Figure 2. The *bont* A gene of the isolated strains in this study and the A2 subtype strains H111880801g and NCTC9837 clustered into one branch confirming the subtype of it.

Based on the result from the MLST database, no existed ST type was found for the seven gene sequences of the 10 strains in the database. These strains were assigned a new MLST type, ST193 [*aroE* (3), *mdh* (3), *aceK* (60), *oppB* (61), *rpoB* (3), *recA* (3), and *hsp* (35)], indicating a (Figure 1A).

### 4.4 Gene cluster of the neurotoxins, antibiotic resistance genes, and virulence factors

**Figure 3:**
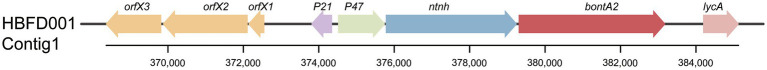
Botulinum neurotoxin-producing gene clusters of the strains represented by HBFD001.

To characterize the neurotoxin gene cluster, the genes located around the *bont* were investigated. The *botR*, *ha17*, *ha34*, and *ha70* genes were all absent in these strains, with the presence of the *ntnh* gene indicating that these strains were not part of the neurotoxin ha cluster. The *orfX*1-3, *P21*, *P47*, and *ntnh* genes were located upstream of *bontA2*, with the *lycA* gene located downstream. This result showed a classic BoNT A2 strain neurotoxin arrangement (Figure 3). Antibiotic resistance genes, including *cfr*, *spw*, and *vat*, were found in all isolated strains, indicating the potential wide range of antibiotic resistance in these strains. In addition to the main virulence BoNT gene, GroEL was detected in all 10 isolated strains, which is not well studied in *C. botulinum*.

## 5 Discussion

BoNT-producing Clostridia are ubiquitous in the environment, leading to frequent contamination of food. Foodborne botulism can cause severe neuroparalytic symptoms and even death, with ingestion of as little as 50 ng of BoNT (Peck, 2009). This study reports a foodborne botulism outbreak caused by homemade pickled eggs contaminated with *C. botulinum* A2 strains in Hebei province, China. One of the patients experienced severe respiratory paralysis and failure, with significant improvement after supportive treatment. The other patient ate more eggs but had milder symptoms, which may be due to the age difference. Both cases had a good prognosis in the subsequent follow-up survey. The best way to treat and shorten the course of the disease is to use antitoxins at an early stage, which requires early clinical diagnosis. No antitoxin was used in this case due to the late diagnosis.

Botulism outbreaks related to pickled eggs contaminated with *C. botulinum* A and B serotypes have been reported in the United States and China (Centers for Disease Control and Prevention, 2000; Miao et al., 2024). We isolated *C. botulinum* A strains not only from patient A’s stool but also from the pickled eggs. No *C. botulinum* was isolated from the stool of Patient B, possibly due to late sampling and low food intake in Patient B. The phylogenetic tree analysis based on whole genome sequencing confirmed that the strains were from the same source, supporting that the source of the outbreak was the pickled eggs. Hebei province is a high-risk area for botulism, with many previous reports of foodborne botulism, including cases involving sausages, pickled eggs, and other meats. However, this is the first reported case of botulism caused by *C. botulinum* A strains from pickled eggs (Shih and Chao, 1986; Hu and Tong, 1988; Miao et al., 2024; Gao et al., 1990). According to previous reports, *C. botulinum* A and B strains have been found in the soil in Hebei, but no genomic sequences are available in published databases to provide direct evidence for tracing the origin of this case (Zhang et al., 2010; Li H. et al., 2022; Dong et al., 2017). Further trace-back efforts are needed to isolate strains from the surrounding environment soil and compare their genomic sequences with the genomic sequences of strains from food and patients.

All the strains belonged to Group I in this study. *C. botulinum* Group I strains are a major cause of the three most common types of botulism in humans: foodborne, infant, and wound botulism (Peck and van Vliet, 2016). Notably, the Botulinum cook (121°C/3 min) process targets *C. botulinum* Group I spores, aiming to ensure the safety of low-acid canned foods. Failures in applying this process to canned or bottled goods, along with temperature abuse of products, have been linked to foodborne botulism outbreaks. In this case, the food was prepared without proper disinfection and stored at excessively high temperatures. These two factors led to the germination of *C. botulinum* spores, their reproduction, and the release of toxins, resulting in subsequent intoxication.

The subtype of the toxin gene in all these 10 isolated *C. botulinum* strains was A2. Two different clusters exist in BoNT-producing strains: the hemagglutinin (ha) toxin gene cluster and the orfX toxin gene cluster (Li T. et al., 2022; Harris et al., 2024; Brunt et al., 2020; Peck, 2009). The ha cluster is found in strains that produce toxin types A1, A5, B, C, D, and G, while the orfX cluster is found in strains that produce toxin types A1 to A4, E, and F (Jacobson et al., 2008a,b; Dover et al., 2013). Here, we found that the isolated A2 strains belonged to the orfX gene cluster, which is consistent with previous studies. In addition to the BoNT gene, another potential virulence factor, GroEL, was found, and its virulence was found to be different in each pathogen (Zhang et al., 2021; Rivera-Ramirez et al., 2022; Zhu et al., 2023). However, its pathogenicity in *C. botulinum* has not been studied yet.

**Figure 4:**
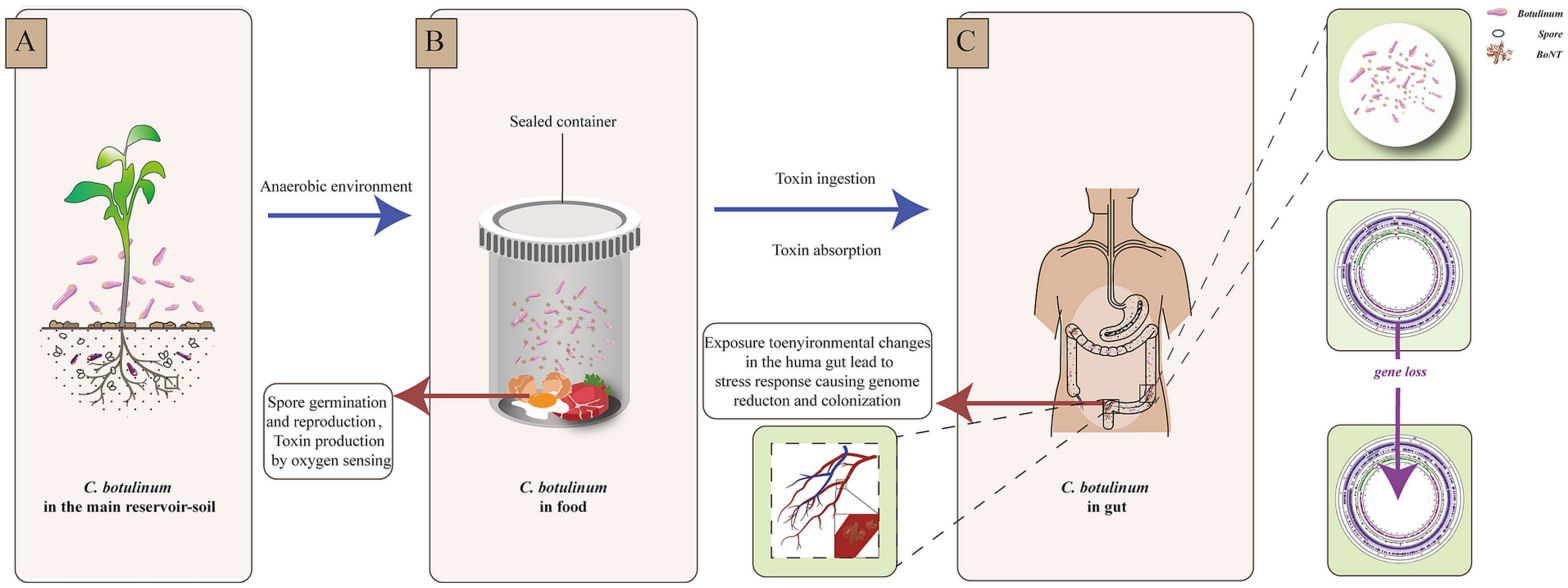
Environmental changes and reaction scheme of *C. botulinum*. **(A)**. *C. botulinum* in the main reservoir—soil. **(B)**. Spore germination and reproduction, toxin production by oxygen sensing in a sealed food container. **(C)**. Stress response of *C. botulinum* in the human gut.

Interestingly, the genome of *C. botulinum* isolated from patient A in this study was smaller than that of *C. botulinum* from the food. This finding was also reported in a study of other botulism outbreaks in Xinjiang Province (Ma et al., 2022). Niche-dependent differential gene loss is assumed to occur when bacteria transition from free-living or facultatively parasitic life cycles to permanent associations with hosts. It has been revealed that habitat is a major factor contributing to genome reduction (Sakharkar and Chow, 2005; Fang et al., 2021). Here, we speculated that the environmental differences and evolutionary pressures were encountered when *C. botulinum* accessed the human intestinal tract. This change could cause a stress response and be more adaptive to the new niche, which might result in gene or DNA loss, as indicated by previous studies on other bacterial species (Hershberg et al., 2007; Perez et al., 1970; Ben Moussa et al., 2023; Pontes et al., 2024). More interestingly, we found that most of the lost genes were related to desiccation/radiation resistance proteins. As this gene was found in more than one strain and in different CDS within the same genome, we hypothesized that the difference was not due to sequencing or assembly. This may be due to a perception of the light and lead to genome loss and possible subsequent reactions, such as colonization (Figure 4). Among the 24 SNPs between the strains from the food and patient, the only functional mutation was found on *cbiH*, which is a putative cobalt-factor III C (17)-methyltransferase. This mutation was a conservative subsection. Other mutations could not be ruled out as being caused by sequencing and assembly.

In conclusion, we reported an outbreak caused by pickled eggs contaminated with a new MLST type *C. botulinum* A2 and confirmed the origin of the contamination through genomic sequence analysis. The toxin, whole genomic sequence, toxin gene, antibiotic resistance, and other virulence factors were analyzed to enhance the understanding of botulism and *C. botulinum.* Considering the high risk of food contamination by Clostridia spores, consumers should be educated on the importance of using clean ingredients and hygienic production practices, as well as the risks associated with these activities and how they can be minimized through effective chilling, chilled storage, and/or fermentation. We should also improve training for clinicians to identify botulism early and provide suggestions for better treatment.

## Data Availability

The datasets presented in this study can be found in online repositories. The names of the repository/repositories and accession number (s) can be found at: https://www.ncbi.nlm.nih.gov/, PRJNA1203909.
